# Changes in composition of colostrum of Egyptian buffaloes and Holstein cows

**DOI:** 10.1186/1746-6148-8-19

**Published:** 2012-03-05

**Authors:** Alaa M Abd El -Fattah, Fawzia HR Abd Rabo, Samia M EL-Dieb, Hany A El-Kashef

**Affiliations:** 1 Dairy Science Department, Faculty of Agriculture, Cairo University, Giza, Egypt

## Abstract

**Background:**

Changes in colostrum composition of Egyptian buffaloes and Holstein cows collected at calving, 6, 12, 24, 48, 72, 96, 120 h and after 14 days of parturition were studied. Total solids, total protein, whey proteins, fat, lactose and ash contents were determined. Macro- and micro-elements, IgG, IgM, IGF-1, lactoferrin and vitamins (A and E) were also estimated.

**Results:**

At calving, the total protein and whey proteins concentration did not differ between buffalo and cow colostrum, while total solids, fat, lactose and ash concentrations were higher in buffalo than in cow colostrum. All components decreased gradually as the transition period advanced except lactose which conversely increased. On the fifth day post-partum, concentration of total protein, whey proteins, fat, ash and total solids decreased by 69.39, 91.53, 36.91, 45.58 and 43.85% for buffalo and by 75.99, 94.12, 53.36, 33.59 and 52.26% for cow colostrum. However, lactose concentration increased by 42.45% for buffalo and 57.39% for cow colostrum. The macro-and micro-elements concentration of both colostrums tended to decline slightly toward normality on the fifth day of parturition. Buffalo colostrum had a higher concentration of vitamin E than cow colostrum during the experimental period. At calving, the concentration of vitamin A in buffalo colostrum was found to be approximately 1.50 times lower than in cow colostrum. The concentrations of IgG, IgM, IGF-1 and lactoferrin decreased by 97.90, 97.50, 96.25 and 96.70% for buffalo and 76.96, 74.92, 76.00 and 77.44% for cow colostrum, respectively after five days of parturition.

**Conclusions:**

There is a dramatic change in buffalo and cow colostrum composition from the first milking until the fifth day of parturition. There are differences between buffalo and cow colostrum composition during the five days after calving. The composition of both colostrums approaches to those of normal milk within five days after parturition.

## Background

Colostrum is vital food for the newborn of all mammals within the first 5-7 days after parturition. Colostrum contains various nutrients (protein, fat, carbohydrate, water- and fat-soluble vitamins and minerals) as well as many biologically active substances such as immunoglobulins, antimicrobial factors, growth factors and others [1-4]. The quality of colostrum has a strong relationship with colostral immunoglobulin level (CIL). The most abundant CIL in the ruminants' colostrum is the IgG, followed by IgM and IgA [5]. Calves are born without blood immunoglobulins (Igs), so they depend on their mother's colostrum in order to obtain immunity [6]. Transference of passive immunity throughout the colostrum is essential for the calf's health and survival during its first days of life [7]. Because colostrum is very important to the newborn, producers must often make provision to have a source of colostrum available if and when the dam does not provide enough high quality colostrum for the calf. In addition, numerous studies suggest that oral administration of bovine colostrum preparations may contribute to human health care both as part of a health promoting diet and as an alternative or supplement to the medical treatment of specified human diseases [8].

The colostrum composition and its quality are influenced by a variety of factors, including maternal age, parity, breed, nutritional status, season, premature parturition, premature lactation, colostral handling factors (pooling colostrum and storage temperature), induction of parturition and health status [9-11].

During transition from colostrum to normal milk, gradual or sometimes sudden changes may occur in composition and properties [12]. Knowing the composition and physical properties of colostrum and post-colostrum secretions will help establish when such milk is suitable for processing and determine the best use for that milk [13].

Several studies have evaluated the changes in the chemical composition of cow colostrum after parturition, but there is not available information about buffalo colostrum which represents approximately 49% [14] of lactating ruminants in Egypt. So, the aim of this study was to follow the changes in colostrum composition of Egyptian buffaloes and Holstein cows after parturition.

## Methods

### Animals and management

Egyptian buffaloes and Holstein cows were selected from open nucleus herd from Cattle Information System of Egypt (CISE) and Technology Centre of Agricultural Production, Faculty of Agriculture, Cairo University. All animals were in the second lactation and their milk production level in the first lactation was 1880 kg/parity for buffalo and 3500 kg/parity for cow. They fed clover misqawi, rice straw and concentrate (16% protein), and housed in free stalls. This study was approved by the University of Cairo Animal Care Committee.

### Collection of colostrum samples

Colostrum samples from 6 buffaloes and 12 cows were collected during the winter seasons of 2009, 2010 and 2011; totalling 18 buffaloes and 36 cows. All samples were analyzed at caving, 6, 12, 24, 48, 72, 96, 120 h and after 14 days of parturition.

### Methods of analysis

Samples were analyzed for total solids, total protein, whey proteins, lactose and ash contents according to [15]. Fat was determined by Gerber method according to [16].

Calcium (Ca), Mg, Na, K, Fe, Cu, Zn, Pb were estimated by inductively coupled plasma atomic emission spectrometry (ICP-AES) using iCAP 6000 Series; Thermo Scientific. Phosphorus was determined colorimetrically according to [17]. Vitamin A was determined according to [18]. Vitamin E was colorimetrically estimated by the method of [19].

A double antibody radioimmunoassay was used to quantify insulin like-growth factor-1 (IGF-1) according to [20]. BioSource IGF-1-RIA-CT kit (Cat. No. KIP1588, BioSource Europe S.A. - Rue de I' Industrie, 8-B- 1400 Nivelles Belgium) was used.

The immunoglobulin G (IgG) and immunoglobulin M (IgM) contents were quantified using Single Radial Immuno Diffusion Technique (SRID) as described by Fahey and Mackelvey [21]. SRID plates containing antibodies to IgG and IgM (Cat. No. RL 200.3, RN 278.3, the Binding Site LTTD^R^, UK) were used.

Lactoferrin content was determined using Enzyme-linked Immunosorbent Assay (ELISA) according to [22]. ELISA plates containing antibodies to bovine lactoferrin (Cat. No.: E0780Bo, Uscn Life Science Inc. Wuhan) were used.

### Statistical analysis

The following two-way analysis model was used to study the effect of breed and time after parturition on the studied traits:

$${\text{Y}}_{\text{ijk}}=\mu +{\text{B}}_{\text{i}}+{\text{T}}_{\text{j}}+{\text{e}}_{\text{ijk}}$$

Where:

Y_ijk_: the observations,   μ: over all mean,

B_i_: the effect of i^th ^breed {i = 1 (buffalo), 2 (cow)},

T_j_: the effect of j^th ^time after parturition; where j = time of sampling (at caving, 6, 12, 24, 48, 72, 96, 120 h and after 14 days of parturition), and

e_ijk_: the random error.

The least significant difference (L.S.D.) test was calculated to compare the significant differences between the mean of different treatments [23]. All statistical calculations were performed using Mstat-c [24]. Results were considered statistically at a significance level of *P *≤ 0.05.

## Results and discussion

### Colostrum gross chemical composition

**Figure 1:**
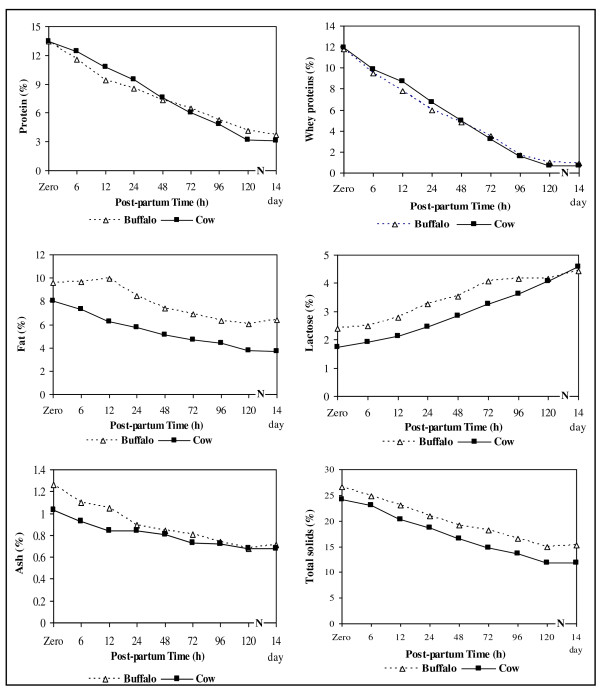
**Changes in gross composition of buffalo and cow colostrum during its transition to normal milk**.

Changes in total protein, whey proteins, fat, lactose, ash and total solids concentration of buffalo and cow colostrum in the first five days and after 14 days of parturition are presented in Figure 1. At calving, the total solids, total protein, whey proteins and fat concentrations of buffalo colostrum were 26.67, 13.46, 11.80 and 9.59% and in cow colostrum were 24.19, 13.45, 11.90 and 8.04%, respectively. Similar results were reported by Kehoe et al., [2], Mechor et al., [25] and Wroński and Sosnowska [26] for cow colostrum and Nawar [27] for buffalo colostrum. Our results showed that total protein and whey proteins concentration did not differ significantly between buffalo and cow colostrum, while fat and total solids were significantly higher in buffalo colostrum. As the transition period advanced, these components in both colostrums decreased gradually. Generally, on the fifth day of parturition, the composition of both colostrums approached to normal milk composition and was similar to that after 14 days of parturition.

At calving, lactose concentration of buffalo colostrum was significantly higher than in cow colostrum (Figure 1). In this respect, [12] found that buffalo colostrum contained 2.70% lactose, while [28] recorded 1.89% for cow colostrum. Lactose concentration increased until the fifth day of parturition. On the 14^th ^day post-partum, lactose concentration of buffalo and cow milk was 4.42% and 4.57%, respectively which was similar to that of the normal milk (Figure 1).

As shown in Figure 1, the first buffalo and cow colostrum had ash percentages of 1.26% and 1.03%, respectively which was similar to that reported by Qyeniyi and Hunter [29] and Toshiyoshi et al., [30] for cow colostrum. At 12 h post-partum, the ash concentration decreased significantly for buffalo (1.045%) and cow (0.84%) colostrum. The ash concentration of both colostrums continued to decrease reaching a value of 0.68%.

### Colostrum macro- and micro-elements

Tables 1 and 2 present the changes in macro- and micro-elements concentration in buffalo and cow colostrum during the first five days and after 14 days of parturition. At calving, cow colostrum had higher concentrations of Mg, Na, K and Zn and lower concentration of P than buffalo colostrum. However, there were no differences between both colostrums in Ca, Fe, Cu and Pb concentrations. The macro- and micro-elements concentration decreased gradually toward normality after five days of parturition. Similar differences in respect to Na during the experimental period (2-28 days of lactation) were reported by Ontsouka et al., [31] for cow colostrum. The opposite trend with regards to K was found by Toshiyoshi et al., [30] for cow colostrum, while [13,31] did not find remarkable change between the initial and final K concentration of cow colostrum.

**Table 1:** Changes in macro-elements content (mg/100 g) of buffalo and cow colostrum during its transition to normal milk (Mean ± SD)

	Ca		Mg		Na		K		P	
Time (h)	Buffalo	Cow	Buffalo	Cow	Buffalo	Cow	Buffalo	Cow	Buffalo	Cow
**At calving**	279.60^c ^± 2.10	281.40^c ^± 2.01	35.52^c ^± 1.00	42.86^a ^± 0.88	150.50^e ^± 1.76	212.80^a ^± 2.20	107.00^g ^± 1.80	179.50^a ^± 1.91	58.00^a ^± 1.00	53.00^de ^± 1.09
**6**	273.20^d ^± 1.90	284.80^b ^± 3.10	33.34^d ^± 1.00	37.76^b ^± 0.80	128.80^g ^± 1.70	213.00^a ^± 2.50	105.30^h ^± 1.71	171.10^b ^± 1.88	56.00^bc ^± 0.80	50.00^f ^± 0.91
**12**	270.60^e ^± 1.80	296.60^a ^± 3.90	29.60^e ^± 1.21	26.90^f ^± 0.51	105.80^i ^± 1.50	201.40^b ^± 1.99	101.30^ij ^± 1.66	160.00^c ^± 1.06	54.00^d ^± 0.50	57.00^ab ^± 1.20
**24**	261.80^f ^± 1.80	245.00^g ^± 2.00	29.20^e ^± 0.90	26.08^g ^± 0.49	81.90^k ^± 1.10	194.20^c ^± 1.88	102.20^i ^± 1.60	123.10^d ^± 1.10	53.00^de ^± 0.50	50.00^f ^± 0.89
**48**	239.80^h ^± 1.73	234.80^i ^± 2.00	25.10^h ^± 0.81	18.11k ± 0.20	76.56^l ^± 1.00	164.30^d ^± 1.45	100.10^j ^± 1.50	119.20^e ^± 0.90	52.00^e ^± 0.30	56.00^bc ^± 0.90
**72**	190.90^k ^± 1.50	199.80^j ^± 2.10	24.12^i ^± 0.79	15.73^l ^± 0.20	56.04^n ^± 0.80	146.80^f ^± 1.50	89.20^k ^± 1.10	100.60^j ^± 1.00	52.00^e ^± 0.80	53.00^de ^± 0.66
**96**	160.10^l ^± 1.60	157.40^m ^± 1.40	19.90^j ^± 0.60	15.10^l ^± 0.10	63.28^m ^± 0.80	120.40^h ^± 1.20	82.63^l ^± 0.64	110.20^f ^± 1.00	54.00^d ^± 1.40	52.07^e ^± 1.00
**120**	140.20^n ^± 1.20	115.20^o ^± 0.80	17.50^k ^± 0.50	14.35^m ^± 0.17	36.44^p ^± 0.50	90.10^j ^± 0.80	79.80^m ^± 0.80	80.60^m ^± 0.65	52.00^e ^± 1.15	55.50^c ^± 1.10
**336**	114.00^o ^± 1.10	93.00^p ^± 0.10	13.46^n ^± 0.30	14.20^m ^± 0.09	38.20^o ^± 0.45	82.10^k ^± 0.64	74.00^n ^± 0.50	65.00^o ^± 0.40	57.00^ab ^± 1.00	53.40^d ^± 0.80
**LSD**	2.362		0.6319		1.068		1.348		1.167	

Means in the same column or row of each element with different superscript letters differ significantly

SD: Standard deviation of the mean

**Table 2:** Changes in micro-elements content (mg/100 g) of buffalo and cow colostrum during its transition to normal milk (Mean ± SD)

	Fe		Cu		Zn		Pb	
Time (h)	Buffalo	Cow	Buffalo	Cow	Buffalo	Cow	Buffalo	Cow
**At calving**	2.36^a ^± 0.06	2.21^a ^± 0.44	0.186^b ^± 0.06	0.186^b ^± 0.01	0.23^b ^± 0.01	0.27^a ^± 0.02	0.028^bcde ^± 0.00	0.042^ab ^± 0.00
**6**	1.50^b ^± 0.07	1.27^c ^± 0.39	0.118^d ^± 0.04	0.204^a ^± 0.01	0.21^cd ^± 0.00	0.20^d ^± 0.02	0.024c^def ^± 0.00	0.035^abc ^± 0.00
**12**	0.77^de ^± 0.06	0.80^d ^± 0.20	0.084^e ^± 0.02	0.178^b ^± 0.01	0.21^cd ^± 0.02	0.22^bc ^± 0.01	0.020c^defg ^± 0.00	0.032^abcd ^± 0.00
**24**	0.76^de ^± 0.05	0.58^ef ^± 0.08	0.082^e ^± 0.02	0.190^ab ^± 0.02	0.20^d ^± 0.02	0.12^h ^± 0.01	0.018^defg ^± 0.00	0.045^a ^± 0.00
**48**	0.66^def ^± 0.04	0.61^def ^± 0.09	0.077^e ^± 0.01	0.176^bc ^± 0.03	0.17^ef ^± 0.01	0.18^e ^± 0.03	0.018^defg ^± 0.00	0.036^abc ^± 0.00
**72**	0.60^def ^± 0.03	0.53^f ^± 0.07	0.060^f ^± 0.01	0.161^c ^± 0.01	0.15^g ^± 0.03	0.16^fg ^± 0.02	0.012^efg ^± 0.00	0.024^cdef ^± 0.02
**96**	0.51^f ^± 0.03	0.54^f ^± 0.06	0.060^f ^± 0.01	0.120^d ^± 0.00	0.13^h ^± 0.01	0.17^ef ^± 0.01	0.010^fg ^± 0.00	0.025^def ^± 0.00
**120**	0.54^f ^± 0.03	0.51^f ^± 0.05	0.052^f ^± 0.00	0.090^e ^± 0.00	0.12^h ^± 0.02	0.15^g ^± 0.01	0.010^fg ^± 0.00	0.010^fg ^± 0.00
**336**	0.53^f ^± 0.01	0.46^f ^± 0.01	0.020^g ^± 0.00	0.060^f ^± 0.00	0.16^fg ^± 0.03	0.17^ef ^± 0.01	0.009^fg ^± 0.00	0.007^g ^± 0.00
**LSD**	0.2163		0.01659		0.01659		0.01659	

Means in the same column or row of each element with different superscript letters differ significantly

SD: Standard deviation of the mean

### Colostrum vitamins (A and E)

Changes in vitamins A and E in buffalo and cow colostrum during the first five days and after 14 days of parturition are shown in Table 3. At calving, the concentration of vitamin A in buffalo colostrum was found to be approximately 1.50 times lower than in cow colostrum. As the post-partum time advanced, the concentration of vitamin A in both colostrums decreased to reach the values of 136.70 and 128.90 IU/100 ml after 14 days in buffalo and cow colostrum, respectively. As for cow colostrum, [32] reported similar results.

**Table 3:** Changes in vitamin A and E content (IU/100 ml) in buffalo and cow colostrum during its transition to normal milk (Mean ± SD)

	Vitamin A		Vitamin E	
Time (h)	Buffalo	Cow	Buffalo	Cow
**At calving**	166.67^g ^± 0.88	250.00^c ^± 2.65	342.00^f ^± 3.30	234.00^j ^± 3.00
**6**	125.00^n ^± 2.65	270.83^b ^± 2.09	402.00^a ^± 4.20	210.20^l ^± 2.31
**12**	145.83^j ^± 1.83	312.50^a ^± 3.28	336.00^g ^± 3.15	198.00^m ^± 1.90
**24**	188.03^e ^± 1.55	187.50^e ^± 1.80	396.00^b ^± 3.90	201.00^m ^± 2.00
**48**	171.90^f ^± 1.73	166.66^g ^± 0.88	387.00^c ^± 3.68	174.00^p ^± 1.60
**72**	156.25^i ^± 2.40	208.33^d ^± 1.46	378.00^d ^± 3.50	186.00^o ^± 1.20
**96**	146.20^j ^± 0.98	159.63^h ^± 1.55	352.97^e ^± 3.60	191.33^n ^± 1.50
**120**	138.20^l ^± 1.18	140.60^k ^± 1.40	341.67^f ^± 2.80	220.67^k ^± 2.90
**336**	136.70^l ^± 0.26	128.90^m ^± 0.90	330.00^h ^± 2.98	318.00^i ^± 5.00
**LSD**	2.12		4.363	

Means in the same column or row of each vitamin with different superscript letters differ significantly

SD: Standard deviation of the mean

Table 3 also shows that the initial value of vitamin E was higher in buffalo than cow colostrum and its concentration in buffalo colostrum decreased after 48 h post-partum and continued to decrease until the end of the experimental period, while vitamin E concentration in cow colostrum had the opposite trend. As for cow colostrum, [32] found that vitamin E concentration decreased during the first five days of parturition.

### Colostrum bioactive components

**Figure 2:**
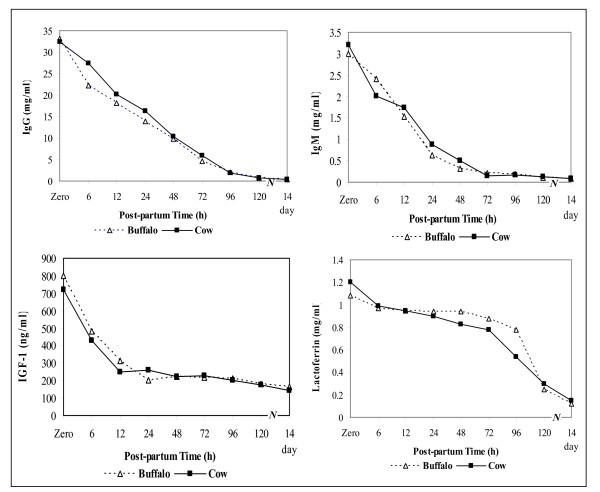
**Changes in IgG, IgM, IGF-1 and lactoferrin concentration of buffalo and cow colostrum during its transition to normal milk**.

The results of IgG, IgM, IGF-1 and lactoferrin of buffalo and cow colostrum are shown in Figure 2. At calving, there were no significant differences in IgG and IgM concentration between buffalo and cow colostrum. The concentrations of IgG and IgM were 33.20 and 3.00 mg/ml in buffalo and 32.33 and 3.20 mg/ml in cow colostrum, respectively. Similar results were published by Mechor et al., [25] for IgG and Quigley et al., [33] for IgM in cow colostrum. The concentrations of IgG and IgM decreased in both colostrums and reached to normal values after five days of parturition and it was observed a sharp decrease in IgG compared to IgM.

At calving, IGF-1 concentration was significantly higher in buffalo colostrum (801.90 ng/ml) than that in cow colostrum (721.90 ng/ml). The concentration of IGF-1 in cow colostrum is in agreement with the results reported by Elfstrand et al., [1]. The concentration of IGF-1 in both colostrums reduced significantly by 40.20% after 6 h of parturition; and by 77.44% for buffalo and by 76.03% for cow colostrum after five days post-partum (Figure 2).

At calving, lactoferrin concentration was significantly lower in buffalo colostrum than that in cow colostrum. The concentration of lactoferrin in the first buffalo colostrum was 1.085 mg/ml and decreased significantly to reach the normal value of 0.123 mg/ml after 14 days of parturition. On the other hand, the first cow colostrum contained 1.196 mg/ml lactoferrin and decreased significantly to 0.30 mg/ml after five days post-partum (Figure 2). Lactoferrin concentration of the first buffalo colostrum is lower than that reported by Nawar [27] and higher than that reported by Mahran et al., [34].

## Conclusions

At calving, the buffalo colostrum has higher concentrations of fat, ash, total solids, lactose, vitamin E, phosphorus and IGF-1 and lower concentrations of Mg, K, Na, Zn, vitamin A and lactoferrin than cow colostrum. On the fifth day of parturition, buffalo colostrum has higher fat, total solids, Ca, Mg and vitamin E and lower Na, P, Cu, Zn and vitamin A concentrations than cow colostrum. The composition of both colostrums approaches to normal milk composition during the first five days after parturition.

## Authors' contributions

AMA and FHRA carried out the study design, participated in data organization, wrote and revised the manuscript; SME participated in data organization, wrote and revised the manuscript; HAE carried out all the experiments, participated in data organization, wrote and revised the manuscript. All authors read and approved the final manuscript.
